# Recent Metabolomics Analysis in Tumor Metabolism Reprogramming

**DOI:** 10.3389/fmolb.2021.763902

**Published:** 2021-11-25

**Authors:** Jingjing Han, Qian Li, Yu Chen, Yonglin Yang

**Affiliations:** ^1^ Department of Anesthesiology, First Affiliated Hospital of Nanjing Medical University, Nanjing, China; ^2^ Division of Infectious Diseases, Taizhou Clinical Medical School of Nanjing Medical University (Taizhou People’s Hospital), Taizhou, China

**Keywords:** metabolomics, metabolic reprogramming, biomarkers, stable isotope resolved metabolomics, drug resistance

## Abstract

Metabolic reprogramming has been suggested as a hallmark of cancer progression. Metabolomic analysis of various metabolic profiles represents a powerful and technically feasible method to monitor dynamic changes in tumor metabolism and response to treatment over the course of the disease. To date, numerous original studies have highlighted the application of metabolomics to various aspects of tumor metabolic reprogramming research. In this review, we summarize how metabolomics techniques can help understand the effects that changes in the metabolic profile of the tumor microenvironment on the three major metabolic pathways of tumors. Various non-invasive biofluids are available that produce accurate and useful clinical information on tumor metabolism to identify early biomarkers of tumor development. Similarly, metabolomics can predict individual metabolic differences in response to tumor drugs, assess drug efficacy, and monitor drug resistance. On this basis, we also discuss the application of stable isotope tracer technology as a method for the study of tumor metabolism, which enables the tracking of metabolite activity in the body and deep metabolic pathways. We summarize the multifaceted application of metabolomics in cancer metabolic reprogramming to reveal its important role in cancer development and treatment.

## Introduction

In recent years, metabolism, as an important link between environmental factors, metabolic small molecules, host genes and diseases, has gained increasing interest regarding its relationship with tumors. Under environmental selection pressures induced by microenvironmental and genetic factors, the inside of the tumor undergoes evolution. Simultaneously, under the control of genotypes, metabolic characteristics of the tumor undergo adaptive changes; this is termed metabolic reprogramming (Zhao et al., 2019). Studies have shown that the tumor microenvironment is often nutritionally deficient; thus, tumor cells reprogram their metabolism and that of the microenvironment to maintain their proliferative ability (Wu and Dai, 2017; Ringel et al., 2020).

Metabolic reprogramming of the tumor microenvironment is considered one of the markers of cancer and an important direction of tumor research (Hanahan and Weinberg, 2011). Continuous developments in high-throughput sequencing and bioinformatics technology have enabled wide use of the combined analysis of metabolomics (Kaushik and DeBerardinis, 2018), genomics (Horn et al., 2019), transcriptomics (Finotello and Trajanoski, 2018), and proteomics (Kim et al., 2019) to determine the etiology and pathogenesis of diseases. Metabolomics is regarded as the final node of various molecular pathways and is considered the ultimate goal of omics research. The rise of metabolomics has allowed considerable progress to be made in understanding the relationship between metabolic regulation and cancer.

**FIGURE 1:**
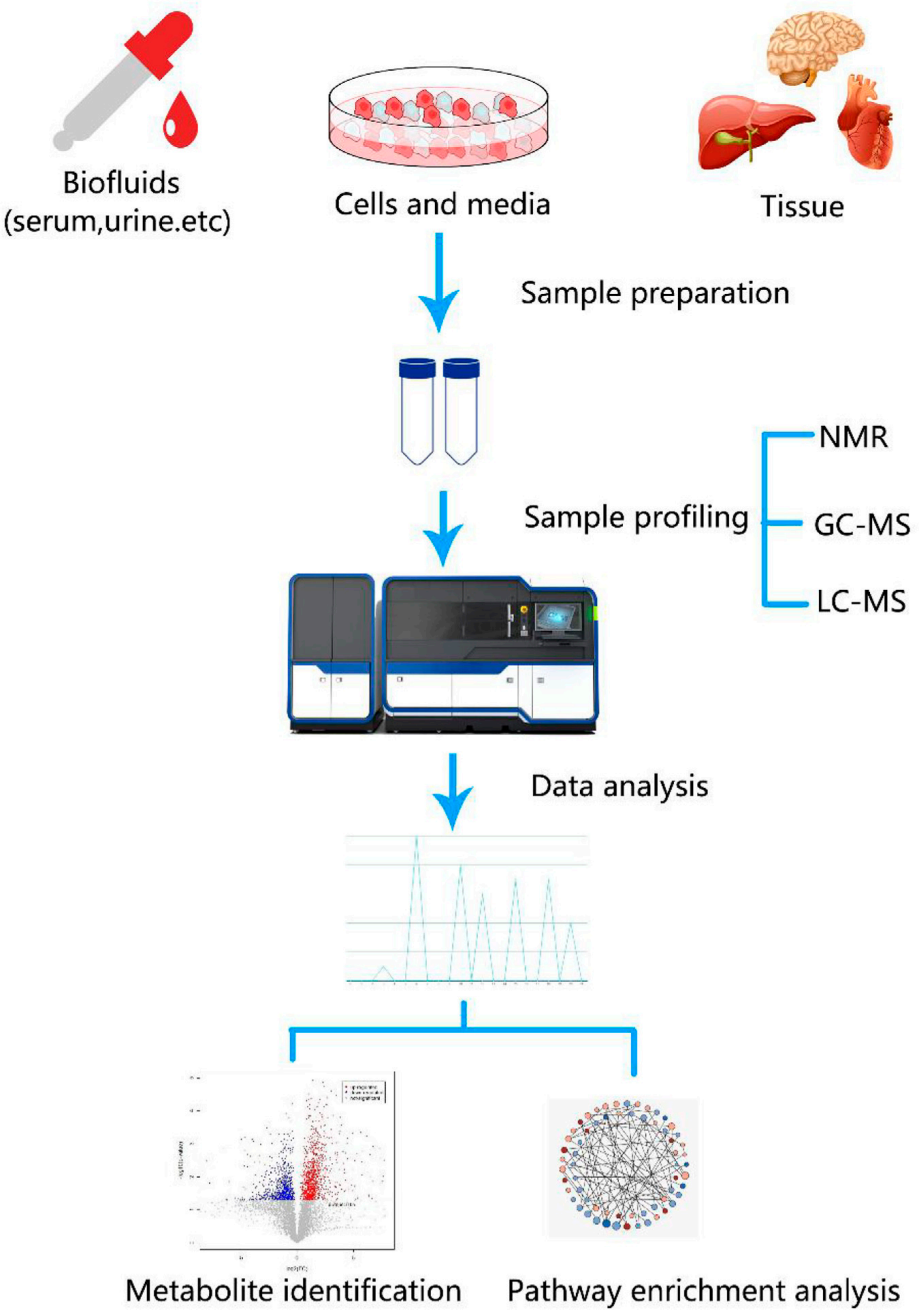
**(A)** Analytical workflow of metabolomics studies. A typical metabolomics study includes experimental design, sample collection, sample profiling, data analysis, and functional interpretation stages. Metabolites from biological fluids, cells, and tissues that differ between tumor and control groups can be detected using metabolomics [e.g., nuclear magnetic resonance (NMR), liquid chromatography-mass spectrometry (LC-MS), and gas chromatography-mass spectrometry (GC-MS)] and data analyses. Discovery of metabolic biomarkers and pathways that are specific to certain cancers benefit cancer research.

Metabolomics techniques are defined as the measurement of the dynamic multiparameter metabolic response of biological systems to various stimuli and genetic changes in specified quantities (Johnson et al., 2016). They primarily involve analyses of metabolites in bodily fluids, cells, and tissues and are usually applied as a valuable means to identify biomarkers (Zampieri et al., 2017). The basic research approach involves measuring metabolites using high-throughput and high-resolution detection technology, acquiring massive datasets, obtaining different metabolites via data analysis, finding metabolic pathways, and explaining their biological significance (Toledo et al., 2017). Metabolomics techniques usually include nuclear magnetic resonance (NMR), liquid chromatography-mass spectrometry (LC-MS), and gas chromatography-mass spectrometry (GC-MS) (Fiehn, 2016; Lane et al., 2019). In addition, the accuracy of qualitative metabolite analyses depends not only on the detection and resolution ability of the mass spectrometer but also on the corresponding metabolite database (Wishart et al., 2013; Johnson et al., 2016). Figure 1 illustrates the basic workflow of metabolomics techniques.

Mass spectrometry-based technology has become the mainstream technology for the analysis of targeted metabolomics pathways. Numerous studies have revealed that differences in small molecular metabolites, such as serum, tissues, urine, and saliva, and changes in corresponding metabolic pathways are closely related to tumor risk, tumor type, and the sensitivity and efficacy of chemotherapy drugs as well as potential drug targets (Wishart 2019). Metabolomics technology could bring a new dimension to tumor metabolism. Several excellent reviews of metabolomics applications in cancer research have been published, which have focused primarily on the search for metabolic biomarkers and investigations on metabolic mechanisms underlying various tumors (Zhang et al., 2014; Xiao and Zhou, 2017). In this review, we first summarize the applications of metabolomics to the three major metabolism pathways of cancers. We then focus on biofluid markers for the early prediction of tumors, metabolomics in cancer drug treatments, and applications of resistance mechanisms. Finally, we introduce the applications of stable isotope tracer technology to the field of metabolomics and offer future directions.

## Metabolomics Analysis of the Three Major Metabolic Pathways of Tumor

**FIGURE 2:**
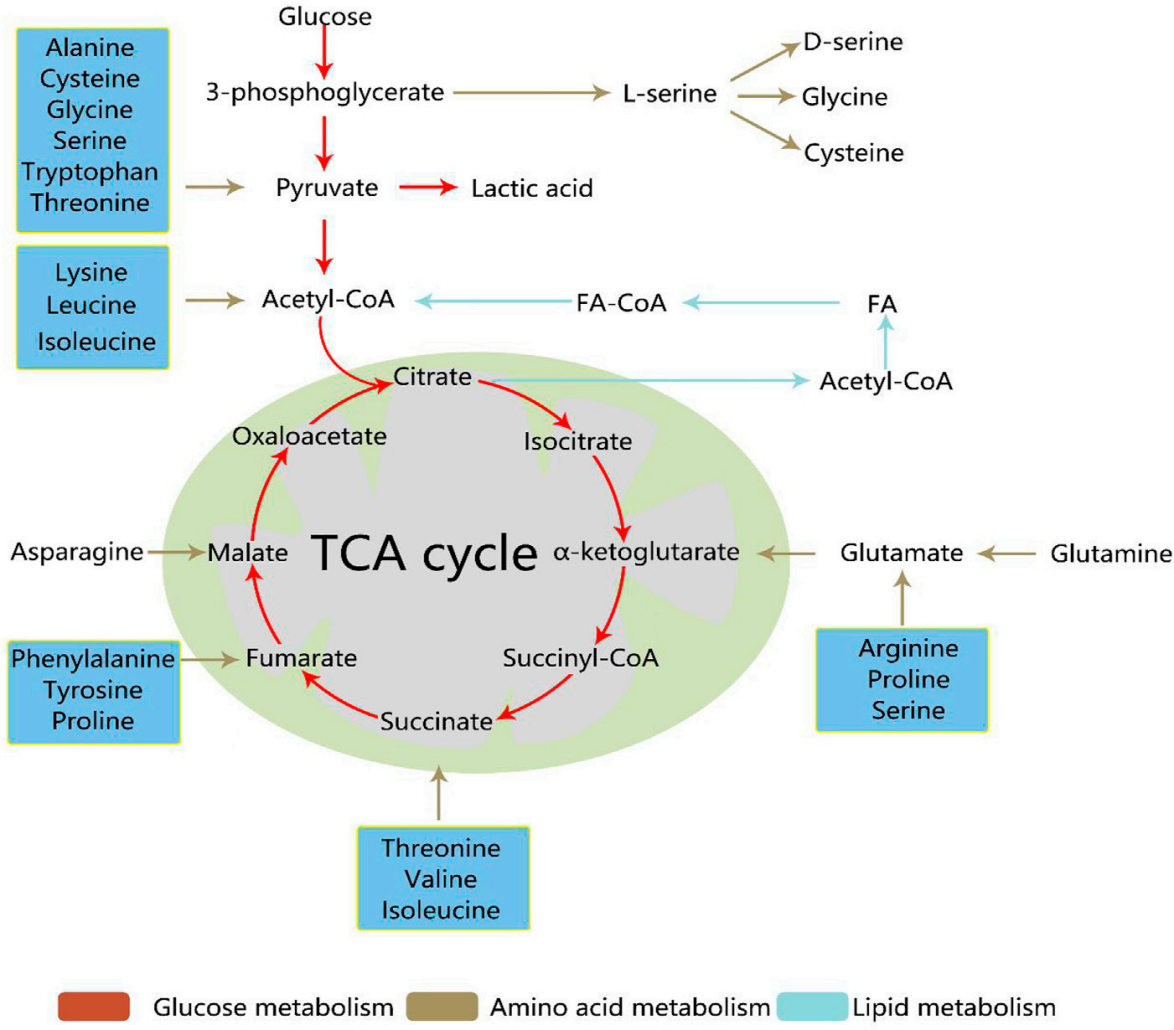
The regulation of the three pathways of cancer cells and their crossover. During cancer development, metabolic reprogramming provides cancer cells the ability to survive and proliferate. Glucose, amino acid, and lipid metabolism are inseparable. Activated glycolysis and impaired aerobic respiration shape the altered glucose metabolism. In addition, deregulated anabolism/catabolism of fatty and amino acids, especially glutamine, serine, and glycine, have been identified to function as metabolic regulators in supporting cancer cell growth. TCA, tricarboxylic acid; FA, fatty acid; acetyl-CoA, acetyl coenzyme A; FA-CoA, fatty acetyl coenzyme A.

During cancer development, metabolic reprogramming provides cancer cells the ability to survive and proliferate. The most famous is the Warburg effect, which suggests that the aerobic glycolysis pathway is closely related to the occurrence of cancer. In addition, deregulated anabolism/catabolism of fatty (FAs) and amino acids, especially glutamine, serine, and glycine, have been shown to function as metabolic regulators in supporting cancer cell growth. The occurrence and development of cancer cells are closely related to the three metabolic pathways. Figure 2 shows the regulation of the three pathways of cancer cells and how they crossover. Metabolomics techniques can be used to supplement tumor metabolism by analyzing the metabolic profiles of different tumors.

### Glucose Metabolism Reprogramming in Cancer Progression

Owing to the need for malignant proliferation, tumor cells exhibit a rapid glycolysis phenomenon in various environments called the Warburg effect. Tumor cells have a high ability to proliferate and a high demand for energy, which often leads to hypoxia in the local tissue microenvironment (Warburg, 1956; Williams et al., 2016). Although glycolysis is not as efficient as aerobic respiration in terms of energy supply, it is 100 times faster than aerobic respiration and provides the amino acids and intermediate metabolites of pentose phosphate essential for highly proliferating cancer cells (Cacciatore and Loda, 2015). Thus, cancer cells weaken or even cease using the mitochondrial aerobic oxidation pathway in favor of the glucose glycolysis pathway for energy, which produces large amounts of lactic acid (Chen et al., 2014). Aerobic glycolysis is a key metabolic feature of the Warburg phenotype and is caused by active metabolic reprogramming that is required to support sustained cancer cell proliferation and malignant progression (Kishton et al., 2016). Glucose metabolism includes not only glycolysis but also other pathways that require glucose. These pathways include pentose phosphate pathway (PPP), hexosamine pathway, glycogenesis. They are all reprogrammed in cancer cells, and this reprogramming can be used to selectively target cancer cells.

High-throughput omics screening has shown that the tumor microenvironment and various cancer-promoting signaling pathways significantly up-regulate the glycolysis process, which results in the Warburg effect. Recent investigations of renal carcinoma cells and tissues from xenografted mice and patients using metabolomics, proteomics, and transcriptomics have unambiguously confirmed that this phenomenon is a key component of metabolic reprogramming (Perroud et al., 2009). As an example of Warburg metabolism, levels of enzymes involved in glycolysis, such as hexokinase-1 pyruvate kinase, and lactate dehydrogenase A were significantly increased in renal carcinoma cells and tissues (Perroud et al., 2006). In a breast cancer study (Dai et al., 2017), researchers cultured MCF-7 and T47D breast cancer cells with different glucose concentrations and found that low glucose concentration significantly inhibited the proliferation of breast cancer cells. Moreover, signal pathway enrichment analysis showed that the Hippo-Yap cell signaling pathway in MCF-7 breast cancer cells was downregulated when the glucose concentration in the culture environment was reduced, whereas the expression of NRF2 pathway-related genes in T47D breast cancer cells was significantly increased (Maldonado et al., 2021). In a recent lung cancer study, researchers found that glucose metabolism disorders may be closely associated with the carcinogenesis of lung cancer, which suggests that glucose metabolism may be a potential therapeutic target for lung cancer (Ding et al., 2019).

Essentially, the Warburg effect in tumor cells is caused by the increased expression of metabolic enzymes related to the glycolysis pathway. In recent years, the regulation of glycolytic metabolic enzymes, especially rate-limiting enzymes, has attracted considerable attention in the field of oncology (Chen et al., 2016). Through metabolomics analysis, researchers confirmed that KRAS gene’s effect on tumor metabolism can be realized through transcriptional regulation of glucose transporters and glycolysis enzymes. And the KRAS activating mutations copy gain creates unique metabolic dependences that can be exploited to selectively target these aggressive mutant KRAS tumors (Kerr et al., 2016). Wong et al. showed that protein arginine N-methyltransferase 6 (PRMT6) regulates aerobic glycolysis in human hepatocellular carcinoma (HCC) through nuclear relocalization of pyruvate kinase M2 isoform (PKM2), a key regulator of the Warburg effect. This research provides a mechanistic link between tumorigenicity of tumors and glucose metabolism (Wong et al., 2020). The tumor microenvironment and various cancer-promoting signaling pathways have been found to significantly upregulate the glycolysis process and thus, providing a variety of potential targets for inhibiting glycolysis in tumor therapy. This has been confirmed in a variety of tumor settings and is associated with poor tumor outcomes (Li et al., 2018; Zhang et al., 2020).

### Lipid Metabolic Reprogramming in Cancer Cells

Tumor cells exhibit metabolic plasticity, which provides a selective advantage for the survival and proliferation of tumor cells in harsh microenvironments, such as hypoxia, acidosis, and malnutrition (Hanahan and Weinberg, 2011). Significant characteristics of lipid metabolism in tumor cells include increased adipogenesis rate and an upregulated mitochondrial fatty acid β-oxidation level (Currie et al., 2013). A variety of tumors have shown similar trends, and various metabolites involved in lipid metabolism have exhibited typical changes. In such microenvironments, tumor cell lipid synthesis is increased (Peng et al., 2018). Lipids provide a large amount of energy for the proliferation of tumor cells to maintain membrane synthesis and other related functions during tumor cell growth. Systemic metabolic alterations associated with increased consumption of saturated fat and obesity are linked with increased risk of prostate cancer progression and mortality. Studies have shown that in primary prostate cancer, dietary saturated fat intake contributes to tumor progression by mimicking MYC over expression, setting the stage for therapeutic approaches involving changes to the diet (Labbé et al., 2019). Lipid metabolomics techniques provide information on lipid changes in various tumor cells (Poczobutt et al., 2016).

Lipid metabolism presents as a network of pathways with flexible feedback loops and crosstalk to meet the increased metabolic needs of cancer cells. Based on multi-omics data of pan-cancer, researchers have found extensive alterations in FAs, arachidonic acid, cholesterol metabolism, and peroxisome proliferator-activated receptor (PPAR)-signaling in different tumors, and lipid metabolism features are shared among tumors with similar tissue origin tumors. Moreover, possible causes of metabolic disorders have been correlated with lipids in tumors from several perspectives, which include somatic mutation, DNA methylation abnormality, and regulation of transcription factors (Hao et al., 2019). For example, hexadecenoic acid, docosahexaenoic acid, heptanoic acid, and β-hydroxybutyrate, are significantly greater in gastric cancer than they are in benign tissue (Stuart et al., 2014). Untargeted metabolomic studies of kidney cancers have shown increased use of FAs by cancer cells (Ganti et al., 2012). Consistent with this finding, FA oxidation inhibitors, such as the PPAR α antagonist GW6471, have been tested in several models of renal clear cell cancer and have been shown to inhibit the growth of related tumor cells (Abu Aboud et al., 2015).

Lipid metabolism can not only affect the growth of tumor cells by metabolic recombination of fatty acids and other molecules, but also regulate the cross-talk between tumor cells and tumor associated stromal cells to modulate the high metabolic needs of the tumor. It has been reported that the liver X receptor (LXR), a lipid activated transcription factor, plays an important role in modulating the TME. Apoptotic tumor cells containing oxysterols activate LXR in macrophages causing suppression of dendritic cell migration and recruitment of neutrophils in tumors resulting in tolerance and immunosuppression (Traversari et al., 2014). Cancer stem cells (CSCs) or tumor-initiating cells (TICs) represent a small group of cancer cells with self-renewing, tumor-initiating, and unique metabolic properties. Unlike most tumor cells, CSCs and TICs are conventionally treated refractory tumors that are the cause of recurrence in cancer patients. Recent advances in metabolomic detection have shown that lipid uptake of new fat to form lipid droplets and induce changes in lipid desaturation and FA oxidation are related to the regulation of CSCs (Brandi et al., 2017). Changes in lipid metabolism not only meet the energy requirements and biomass production of CSCs but also activate several important carcinogenic signaling pathways, including Wnt/β-catenin and Hippo/YAP signaling pathways (Wang et al., 2016; Yi et al., 2018). It has been suggested that lipid metabolism in tumor cells and its role in tumor progression and metastasis have attracted more and more attention.

### Amino Acid Metabolism Reprogramming in Tumor Growth

Cancer cells’ need for amino acids increases in order to sustain their rapid proliferation. In addition to their use in protein synthesis, amino acids are increasingly being studied as metabolites and regulators that support cancer cell growth (Wen et al., 2018). Multiple amino acids have been confirmed to be valuable in the identification of potential biomarkers and understanding the pathogenesis of various malignant tumors. Among these, the study of glutamine, serine, and glycine has been a primary focus (Jung et al., 2014; Yip-Schneider et al., 2019). Amino acid uptake, steady-state levels, and catabolism are all elevated in the leukemia stem cell (LSC) population (Jones et al., 2018). Changes in the amino acid metabolic spectrum are correlated with the occurrence of gastric cancer, and the amino acid metabolic pathway is abnormal in gastric cancer patients, as shown by the significant correlation between the levels of alanine and arginine and cancer T stage (Chen et al., 2010).

Increased glutamine metabolism is a commonly observed metabolic change in cancer, and glutamine is second only to glucose in importance as a nutrient in cancer. As the most abundant free amino acid, glutamine is involved in a series of energy generation, macromolecular synthesis, and signal transmission pathways of cancer cells by providing nitrogen and carbon atoms (Coloff et al., 2016; Kappler et al., 2017). Glutamine can synthesize a variety of other amino acids to participate in the tricarboxylic acid (TCA) cycle. Moreover, glutamine-derived fumarate, malate, and citrate increase significantly when glucose is deprived, which suggests that glutamine drives the glucose-independent TCA cycle (Spinelli et al., 2017). Increasing glutamine for mitochondrial-dependent bioenergy production and cell biosynthesis is a key feature of many tumor cells (Wang et al., 2019). In a study that analyzed differences in metabolic profiles between gastric cancer (GC) and gastric ulcer (GU), researchers used LC-MS-based plasma metabolic analysis and found that plasma ornithine levels were higher, and plasma glutamine, histidine, arginine, and tryptophan levels were lower in GC patients than in GU patients (Jing et al., 2018). Several independent studies have also shown higher utilization of glutamine in renal clear cell carcinoma compared with that in normal renal tissue (Wettersten et al., 2015; Hakimi et al., 2016).

Other amino acids, such as aspartic acid and arginine, are involved in the reprogramming of amino acid metabolism in cancer. In a study that investigated significant metabolomic changes in plasma in the early and late stages of 4T1 metastatic breast cancer in mice, the plasma arginine concentration was higher during the early stage of metastasis but gradually decreased, and the urine-arginine/arginine ratio increased in the late stages (Kus et al., 2018). This is consistent with the activation of the arginine metabolic pathway in cancer. Xie et al. used a combined liquid and gas chromatography technology to compare and the plasma metabolic spectrum in breast cancer patients and found that aspartic acid concentration was significantly negatively correlated with the risk of breast cancer (Xie et al., 2015). In addition, low serum aspartic acid concentrations were found to be unique in breast cancer patients, and no significant changes were observed for the serum aspartic acid levels of other malignant tumors, such as gastric and colorectal cancer (Zhang et al., 2020). Aspartic acid is a major neurotransmitter that is known to inhibit tumor cell proliferation, and it may induce tumor cell death through the Akt pathway (Chen et al., 2020). Antimetabolites that interrupt amino acid synthesis have also been developed and are undergoing clinical trials as cancer therapeutics (Tabe et al., 2019).

## Metabolomic Markers in Cancer Progression

With the comprehensive development of modern molecular biology, many new tumor markers carry important clinical value for the early diagnosis and screening of malignant tumors; however, current routine tumor markers lack sensitivity and specificity for the early diagnosis of tumors. As an emerging omics technology, metabolomics mainly involves the study of small molecular metabolites (i.e., those less than 1,500 Da) and reflects a series of small changes in the body at the level of metabolites, which is suitable for the diagnosis of diseases. Many scholars have used a variety of detection techniques to conduct research on the early diagnosis and treatment prediction of tumors (Srivastava and Creek, 2019). Metabolomics approaches are used to identify and validate metabolic biomarkers that accurately and sensitively diagnose tumor field progression and metastasis in a clinical setting. Furthermore, such efforts can be left to clinicians with appropriate time frames to promote early and effective therapeutic interventions, which will significantly improve the 5-years survival rate of tumor patients. We summarize the major findings of previous tumor blood metabolome studies (Table 1).

**TABLE 1:** Metabolites in biofluid samples of cancer and non-cancer groups.

Year	Sample types	Tumor types	Patients/animal models	Method	Discriminant metabolites or findings	Related metabolic pathways	Ref
2016	plasma	Papillary thyroid microcarcinoma	patients with cancer (n = 26) from healthy controls (n = 17)	NMR	Elevated levels of glucose, mannose, pyruvate and 3-hydroxybutyrate in plasma, are involved in the metabolic alterations in papillary thyroid microcarcinoma	Glycolysis, amino acid	Lu et al. (2016)
2016	plasma	Lung and liver cancer	lung (n = 50) and liver cancer patients (n = 50)	LC-MS	two values was discovered to identify lung and liver cancer, which were the product of the plasma concentration of putrescine and spermidine; and the ratio of the urine concentration of S-adenosyl-l-methionine and N-acetylspermidine	The pathways of polyamines metabolome	Xu et al. (2016)
2020	plasma	Pancreatic cancer	patients with pancreatic cancer (n = 60) from healthy controls (n = 60)	LC-MS	The top 10 ranked differential metabolites were precisely aligned as glycocholic acid, agmatine, melatonin, beta-sitosterol, sphinganine, hypoxanthine, spermidine, hippuric acid, creatine and inosine.new metabolite biomarkers in plasma (creatine, inosine, beta-sitosterol, sphinganine and glycocholic acid) can be used to readily diagnose pancreatic cancer in a clinical setting	purine metabolism, glycine and serine metabolism, arginine and proline metabolism, steroid biosynthesis, sphingolipid metabolism and bile metabolism	Luo et al. (2020)
2019	plasma	Pancreatic cancer	patients with pancreatic cancer (n = 22) from healthy controls (n = 40)	LC-MS	About 270 lipids belonging to 20 lipid species were found significantly dysregulated. LysoPC 22:0, PC (P-14:0/22:2) and PE (16:0/18:1) are all associated with tumor stage, CA19-9, CA242 and tumor diameter. What’s more, PE (16:0/18:1) is also found to be significantly correlated with the patient’s overall survival	lipids	Tao et al. (2019)
2014	plasma	Oral squamous cell carcinoma	Patients with locally advanced OSCC(n = 105)	GC-MS	Chemotherapy leads to up-regulation of fatty acids, steroids, and antioxidant substances. Lactate, glucose, glutamate, aspartate, leucine, and glycerol are associated with efficacy of induction chemotherapy. Lactate, glutamate, and aspartate can precisely predict the suitability and efficacy of induction chemotherapy	Glycolysis, amino acid, fatty acid	Ye et al. (2014)
2019	plasma	Breast cancer	1,624 first primary incident invasive breast cancer cases and 1,624 matched controls	LC-MS	There were significant differences in lysoPCs in breast cancer patients. LysoPC aaC18:0 was negatively associated with the risk of breast cancer, while higher concentrations of phosphatidylcholine PC ae C30:0 were associated with an increased risk of breast cancer	lysoPCs	His et al. (2019)
2018	plasma	Pancreatic cancer	pancreatic ductal adenocarcinoma (n = 271), chronic pancreatitis (n = 282), liver cirrhosis (n = 100) or healthy as well as non-pancreatic disease controls (n = 261)	GC-MS	Proline, Sphingomyelin (d18:2, C17:0), Phosphatidylcholine, Isocitrate (C18:0, C22:6), Sphinganine-1-phosphate (d18:0), Histidine, Pyruvate, Ceramide (d18:1, C24:0), Sphingomyelin (d17:1, C18:0) and CA19-9 formed a biomarker signature. The biomarker signature could be identified as a differential diagnosis between pancreatic ductal adenocarcinoma (PDAC) and chronic pancreatitis (CP)	complex lipids, fatty acids and related metabolites	Mayerle et al. (2018)
2017	Urine	Prostate cancer	64 prostate cancer patients and 51 individuals diagnosed with benign prostate hyperplasia	NMR	Branchedchain amino acids, glutamate, pseudouridine, glycine, P = 0.015; dimethylglycine, fumarate and 4-imidazole- acetate were able to distinguish between prostate cancer and benign prostate hyperplasia (BPH)	TCA cycle of glucose metabolism	Pérez-Rambla et al. (2017)
2012	Urine	Kidney cancer	(Group A: 29 cancer patients, 33 controls; Group B: 6 cancers,6 controls)	GC-MS	Results showed differential urinary concentrations of several acylcarnitines as a function of both cancer status and kidney cancer grade, with most acylcarnitines being increased in the urine of cancer patients and in those patients with high cancer grades	acylcarnitines	Ganti et al. (2012)
2011	Urine	Bladder cancer	27 bladder cancer (BC) patients and 32 healthy controls	LC-MS	Cancer patients have elevated levels of acetyl carnitine and adipate in their urine. Carnitine C9:1 and component I, were combined as a biomarker pattern	Fatty acid and carnitine metabolism	Huang et al. (2011)
2020	Urine	Breast cancer	patients with breast cancer (n = 56) and benign breast tumors (n = 22), as well as from healthy females (n = 20)	GC-MS	1-methyl adenosine (1-MA), 1-methylguanosine (1-MG) and 8-hydroxy-2′-deoxyguanosine (8-OHdG) levels were significantly elevated in the early stages of breast cancer, but no significant differences were observed between the benign tumor group and the healthy group	nucleoside metabolomes	Omran et al. (2020)
2013	Urine	Ovarian cancer	40 preoperative epithelial ovarian cancer (EOC) patients, 62 benign ovarian tumor (BOT) patients, and 54 healthy controls	LC-MS	The concentrations of some urinary metabolites of 18 postoperative EOC patients among the 40 EOC patients changed significantly compared with those of their preoperative condition, and four of them suggested recovery tendency toward normal level after surgical operation, including N4-acetylcytidine, pseudouridine, urate-3-ribonucleoside, and succinic acid	nucleotide metabolism, histidine metabolism, tryptophan metabolism, mucin metabolism	Zhang et al. (2013)
2019	Urine	Lung cancer	lung cancer (n = 32) and healthy controls (n = 29)	GC–MS	Six metabolites were altered in urine (l-glycine, phosphoric acid, isocitric acid, inositol, palmitic acid and stearic acid) and four metabolites (l-glycine, phosphoric acid, isocitric acid and inositol) were decreased from patients with cancer, indicating a strong, unified marker of lung cancer pathology	Fatty acid and glucose metabolism	Callejón-Leblic et al. (2019)
2010	Saliva	Oral, breast and pancreatic cancer	69 oral, 18 pancreatic and 30 breast cancer patients, 11 periodontal disease patients and 87 healthy controls	CE-TOFMS	They identified 57 principal metabolites that can be used to accurately predict the probability of being affected by each individual disease. Patients with oral cancer had significantly higher levels of salivary polyamines compared to the control group, and taurine and piperidin were identified as oral cancer-specific metabolites, providing promising markers for oral cancer screening	Polyamines and amino acid metabolism	Soini et al. (2010)
2016	Saliva	Oral cancer	patients with oral cancer (n = 24) and healthy controls (n = 44)	CE-TOFMS	In total, 85 metabolites in tumor and 45 metabolites in saliva were identified to be significantly different between oral cancer and controls, and the combination of S-adenosylmethionine and pipecolate can discriminate oral cancers from controls	metabolites in the urea cycle and one carbon cycle	Ishikawa et al. (2016)
2017	Saliva	Oral squamous cell carcinoma	22 patients with oral squamous cell carcinoma (OSCC) and 21 healthy controls	CE-TOFMS	A total of 25 metabolites were revealed as potential markers to discriminate between patients with OSCC and healthy controls	Choline and metabolites of the BCAA cycle	Ohshima et al. (2017)
2019	Saliva	Breast cancer	101 patients with invasive carcinoma of the breast, 23 patients with ductal carcinoma *in situ*, and 42 healthy controls	LC-MS	The levels of polyamines in the saliva of breast cancer patients were significantly increased. In addition, polyamines and their acetylated forms were elevated invasive carcinoma of the breast only	Polyamine metabolism	Murata et al. (2019)
2018	Saliva	Pancreatic cancer	patients with PC (n = 39), those with chronic pancreatitis (CP, n = 14), and controls (C, n = 26)	CE-TOFMS	Polyamines, such as spermine, N₁-acetylspermidine, and N₁-acetylspermine, showed a significant difference between patients with PC and those with C, and the combination of four metabolites including N₁-acetylspermidine showed high accuracy in discriminating PC from the other two groups	Polyamine metabolism	Asai et al. (2018)
2012	CSF	Malignant gliomas	10 patients presenting malignant gliomas and seven control patients that did not present malignancy	LC-MS	One subtype contained metabolites rich in citric acid cycle components that distinguished the metabolic characteristics of patients with malignant glioma from those in the control group. Newly diagnosed patients were classified into different subtypes and showed low levels of metabolites involved in tryptophan metabolism, which may indicate a loss of inflammatory features	Metabolites from the citric acid cycle, gluconeogenesis, and pyrimidine metabolism, urea cycle	Locasale et al. (2012)
2013	CSF	Glioma	32 patients with histologically confirmed	GC–MS	The citric and isocitric acid levels were significantly higher in the glioblastoma (GBM) samples than in the grades I-II and grade III glioma samples. In addition, the lactic and 2-aminopimelic acid levels were relatively higher in the GBM samples than in the grades I-II glioma samples. The CSF levels of the citric, isocitric, and lactic acids were significantly higher in grade I-III gliomas with mutant isocitrate dehydrogenase (IDH) than in those with wild-type IDH.	Metabolites from the aerobic glycolysis	Nakamizo et al. (2013)
2020	CSF	Medullo-blastoma (MB)	8 patients diagnosed with recurrent MB and 7 healthy controls	LC-MS	The up-regulation of tryptophan, methionine, serine and lysine, which have all been described to be induced upon hypoxia in CSF. While cyclooxygenase products were hardly detectable, the epoxygenase product and beta-oxidation promoting lipid hormone 12,13-DiHOME was found to be strongly up-regulated	Lipid and amino acid metabolism	Reichl et al. (2020)
2020	CSF	different types of brain tumors	A cohort of 163 histologically-proven patients with brain disorders	LC-MS	A total of 508 ion features were detected by the LC-Q/TOF-MS analysis, of which 27 metabolites were selected as diagnostic markers to discriminate different brain tumor types	Amino acids and citrate metabolism	Wang et al. (2020)

NMR, nuclear magnetic resonance; GC-MS, gas chromatography-mass spectrometry; LC-MS, liquid chromatography-mass spectrometry; CE-TOFMS, capillary electrophoresis time-of-flight mass spectrometry; CSF, cerebrospinal fluid.

### Blood Biomarkers

Because blood is a readily available biological specimen, blood biomarker studies account for the majority of metabolomics and tumor studies. The application of metabolomics technology has led to significant breakthroughs in the discovery of biomarkers for a variety of tumors, which include pancreatic, liver, lung, and breast cancers (Ye et al., 2014; Xu et al., 2016; His et al., 2019). For instance, Lu et al. used NMR spectroscopy to screen metabolic changes in thyroid tissues and plasma from papillary thyroid microcarcinoma patients respectively. The results revealed reduced levels of fatty acids and elevated levels of several amino acids in thyroid tissues (Lu et al., 2016). This work illustrates that the metabolomics approach is capable of providing more sensitive diagnostic results and more systematic therapeutic information for all kinds of tumor.

In the case of pancreatic cancer, researchers have identified five new metabolic biomarkers (creatine, inosine, beta-sitosterol, sphinganine and glycocholic acid) that can be used for the clinical diagnosis of pancreatic cancer by comparing plasma metabolomics between patients with pancreatic cancer (n = 60) and healthy controls (n = 60) (Luo et al., 2020). Subsequently, a large prospective study comparing the lipid metabolomics of serum exosomes between pancreatic cancer patients and healthy controls showed that approximately 270 lipids were significantly dysregulated. Further analysis of the correlation between these abnormal lipids and other phosphatidylcholine (PC)-related factors showed that LysoPC 22:0, PC (14:0/22:2), and PE (16:0/18:1) were correlated with tumor stage, CA19-9, CA242, and tumor diameter. In addition, PE (16:0/18:1) was significantly associated with overall survival (Tao et al., 2019). Currently, non-invasive diagnostic tests can only distinguish pancreatic ductal adenocarcinoma (PDAC) from chronic pancreatitis (CP) in approximately two-thirds of patients. Using untargeted metabolomics techniques, Mayerle demonstrated that a biomarker signature (nine metabolites and an additional CA19-9) could be identified as differential diagnoses of PDAC and CP (Mayerle et al., 2018). In addition, inflammatory metabolites identified by serum metabolomics can also stratify tumor and improve diagnosis of patients with aggressive tumor (Cacciatore et al., 2021). The discovery of metabolomics for tumor blood biomarkers can be extended to various aspects, which include the discovery of significantly different metabolites, changes in the tumor metabolic spectrum, and direct identification of tumors and inflammation.

### Urine Biomarkers

The non-invasive nature of urine biomarkers makes them particularly suitable for a wide range of screening purposes, especially for measuring urine from asymptomatic high-risk groups to distinguish between those who may and may not be carrying a disease. Accurate and effective analyses of urine metabolites offer promise for their applications to provide a deeper understanding of tumor pathology and eventually, clinical transformations (Pérez-Rambla et al., 2017; Dinges et al., 2019).

In urological tumors, Ganti used GC-MS to measure carnitine in two different laboratories (Laboratory A: 29 cancer patients and 33 controls; Laboratory B: 6 cancer patients and 6 controls) and showed that differences in urinary concentrations of several acylcarnitines were a function of cancer status and renal cancer grade. Most cancer patients and those with high-grade cancers have increased acylcarnitine in their urine (Ganti et al., 2012). In a bladder cancer study, researchers reported elevated levels of acetylcarnitine and adipate in the urine of cancer patients, which suggested dysregulation of FA metabolism. Changes in the mitochondrial TCA cycle and energy metabolism or the excessive production of acetyl-coenzyme A (acetyl-CoA) lead to changes in acetylcarnitine levels (Huang et al., 2011). Under normal physiological conditions, lipid in urine is limited; thus, the increase in lipid markers in urine is a clear indication of tumorigenesis, especially in the urinary system. Moreover, in non-urinary tumors, such as liver, stomach, cervical, and breast cancers, differential metabolites in urine distinguish cancer patients from healthy controls (Dinges, Hohm et al., 2019). Omran used GC-MS to detect urine metabolites from breast cancer patients and compared these with samples from patients with benign breast tumors and healthy women. Results suggested that 1-methyl adenosine (1-MA), 1-methylguanosine (1-MG), and 8-hydroxy-2′-deoxyguanosine (8-OHdG) levels were significantly elevated in the early stages of breast cancer; however, no significant differences were observed between the benign tumor group and the healthy group (Omran et al., 2020). Urine also plays a role in the uniqueness of biomarkers for specific cancer types (Zhang et al., 2013; Callejón-Leblic et al., 2019).

### Salivary Biomarkers

In addition to blood and urine, other biological fluid biomarkers are available for the early diagnosis of specific cancers. Saliva is a biological fluid made up of more than 99% water and less than 1% of proteins, electrolytes, and other low-molecular-weight components (Soini et al., 2010). Saliva plays a key role in lubrication, chewing, swallowing, and digestion. It protects the integrity of oral tissue and also provides clues for local and systemic diseases and conditions (Abraham et al., 2012). In 2010, using capillary electrophoresis time-of-flight mass spectrometry (CE-TOFMS), Sugimoto found that saliva metabolites were embedded with cancer-specific signals. They performed a comprehensive metabolite analysis of saliva samples from patients with oral cancer and periodontal disease and healthy controls. Patients with oral cancer had significantly higher levels of salivary polyamines compared with the control group, and taurine and piperidine were identified as oral cancer-specific metabolites, which may be promising markers for oral cancer screening (Sugimoto et al., 2010). To explore applications of salivary metabolite biomarkers in oral cancer screening, hydrophilic metabolites in the saliva and tumor tissues of patients with oral cancer were analyzed using CE-TOFMS. In total, 85 metabolites in tumors and 45 metabolites in saliva were identified to be significantly different between oral cancer patients and controls, and the combination of S-adenosylmethionine and pipecolate discriminated oral cancer patients from controls (Ishikawa et al., 2016).

Since then, several studies have shown that the combination of saliva and tumor metabolomics is beneficial for the identification of salivary metabolite biomarkers and the screening of noninvasive oral cancers (Ohshima et al., 2017). In addition to salivary metabolomics of oral cancer, metabolite profiles of saliva in other cancers and diseases have been analyzed (Murata et al., 2019). Great progress has been made in the clinical application of salivary biomarkers. Several biomarkers for systematic cancer detection have been identified and validated at preclinical levels (Asai et al., 2018). The discovery of saliva biomarkers also has special significance for noninvasive identification and recognition of tumors.

### Cerebrospinal Fluid Biomarkers

Cerebrospinal fluid (CSF) is a biological fluid that is most likely to be affected by central nervous system dysfunction, and its analysis can better reflect inherent neurological and biochemical changes (Crews et al., 2009). In 2012, Locasale et, al. first analyzed the metabolic profiles of 10 patients with malignant glioma and seven control patients with non-malignant glioma using a targeted mass spectrometry metabolomics platform and reported a significant association between CSF metabolites and glioma malignancy (Locasale et al., 2012). The study also provided the first global assessment of the polar metabolic composition of CSF associated with malignancies and demonstrated that data acquired using mass spectrometry technology may have sufficient predictive power for the identification of biomarkers and classification of neurological diseases.

In recent years, several studies have also confirmed that the use of untargeted metabolomics techniques to analyze metabolic characteristics of different brain tumors in clinical CSF samples enables reliable identification of significant metabolic differences between different brain tumors, which offers significant promise for diagnoses of brain tumors (Nakamizo et al., 2013; Reichl et al., 2020; Wang et al., 2020). More metabolites, including tricarboxylic acid cycle products, tryptophan and methionine, were also found in the cerebrospinal fluid of gliomas and metastatic tumors (Ballester et al., 2018). Brain tumors are often associated with ischemic necrosis and inflammatory responses. Inflammation-related markers can be detected in the cerebrospinal fluid, which may aid the diagnosis of CNS tumors. Elevated levels of inflammatory markers, such as interleukin-10 and soluble interleukin-receptor, were found in the cerebrospinal fluid of primary CNS lymphoma (Geng et al., 2021). Specific cerebrospinal fluid biomarkers could help avoid high-risk biopsy operations and unnecessary craniotomy, and even guide preoperative surgical planning.

## Metabolomics and Oncology Drugs

### Application of Metabolomics in the Evaluation of Tumor Drug Efficacy

The main purpose of drug therapy of tumors is to control the growth of the tumor and improve the quality of life of patients. Selecting the most effective anti-tumor drugs has become a top priority. In clinical practice, metabolomics can be used to detect body or cell metabolites that reflect the effects of anti-tumor drugs on the body or cells to improve the efficacy of drugs and reduce avoidable adverse reactions.

Kim et al. used NMR to investigate metabolic alterations following adriamycin (ADR) treatment for gastric adenocarcinoma. After human gastric adenocarcinomas were implanted into mice, ADR was intraperitoneally administered for 5 days, and urine was collected on days 2 and 5. Results showed that the levels of trimethylamine oxide, hippurate, and taurine decreased in the tumor model and increased following ADR treatment. In addition, the levels of 2-oxoglutarate, 3-indoxylsulfate, trigonelline, and citrate, which all increased in the tumor model, significantly decreased to those of normal controls following ADR treatment (Kim et al., 2013). In a plasma metabolomics study of 54 patients with colorectal cancer who received capecitabine before and after treatment, it was found that the content of low-density lipoprotein-derived lipids was positively correlated with drug toxicity during treatment (Backshall et al., 2011).

Furthermore, using untargeted lipidomics and quantitative polymerase chain reaction, Zhang et al. identified distinct features of lipid metabolism in imidazole ketone erastin (IKE)-induced ferroptosis and demonstrated that IKE slows tumor growth (Zhang et al., 2019). Once drugs act on the body, changes in genes and/or proteins can impact changes in terminal metabolites, which can be reflected at the metabolic level. Therefore, the early efficacy of drugs can be assessed using metabolomics, which enables medication regimens to be adjusted.

### Application of Metabolomics in the Evaluation of Drug Resistance in Cancer

The metabolic pattern of tumor cells changes following the development of drug resistance. The same drug can produce different metabolic changes in sensitive and drug-resistant cells (Zhang et al., 2016). Therefore, metabolomics can be used to detect metabolic changes in cells and their response to drugs to determine whether tumor cells are resistant to drugs and monitor drug resistance as early as possible. As a fast, simple, and effective method, metabolomics uses a multivariable and dynamic method to evaluate metabolic results across a variety of physiological and pathological states, which allows the prediction and assessment of patients’ sensitivity and drug resistance to chemotherapy.

Metabolomics can be used to distinguish between platinum resistance and metabolite levels. Poschner et al. used LC-MS to characterize the levels of steroids, active estrogen, and sulfated or aldehyde glucose during the development of platinum resistance in ovarian cancer and found that these metabolites are highly expressed in carboplatin-sensitive cells (Poschner et al., 2020). In a study of non-small cell lung cancer (NSCLC), cisplatin-resistant cells were more sensitive to nutrient deprivation than were sensitive cells, and adding glutamine to cisplatin-resistant cells restored cell death due to nutrient deprivation by increasing the intracellular nucleotide concentration. Therefore, cisplatin-resistant patients can improve efficacy by combining drugs, such as 5-fluorocrail, that target nucleoside metabolism (Obrist et al., 2018). The metabolic pattern analysis of cancer patients can also find the metabolic differences between drug-resistant patients and drug-sensitive patients, so as to monitor the drug resistance of patients as early as possible and carry out follow-up treatment (Jiye et al., 2010). Metabolomics has made great strides in the study of drug resistance genes in tumors.

## Metabolic Flux Analysis and Fluxomics in Cancer Metabolism Explorations

**FIGURE 3:**
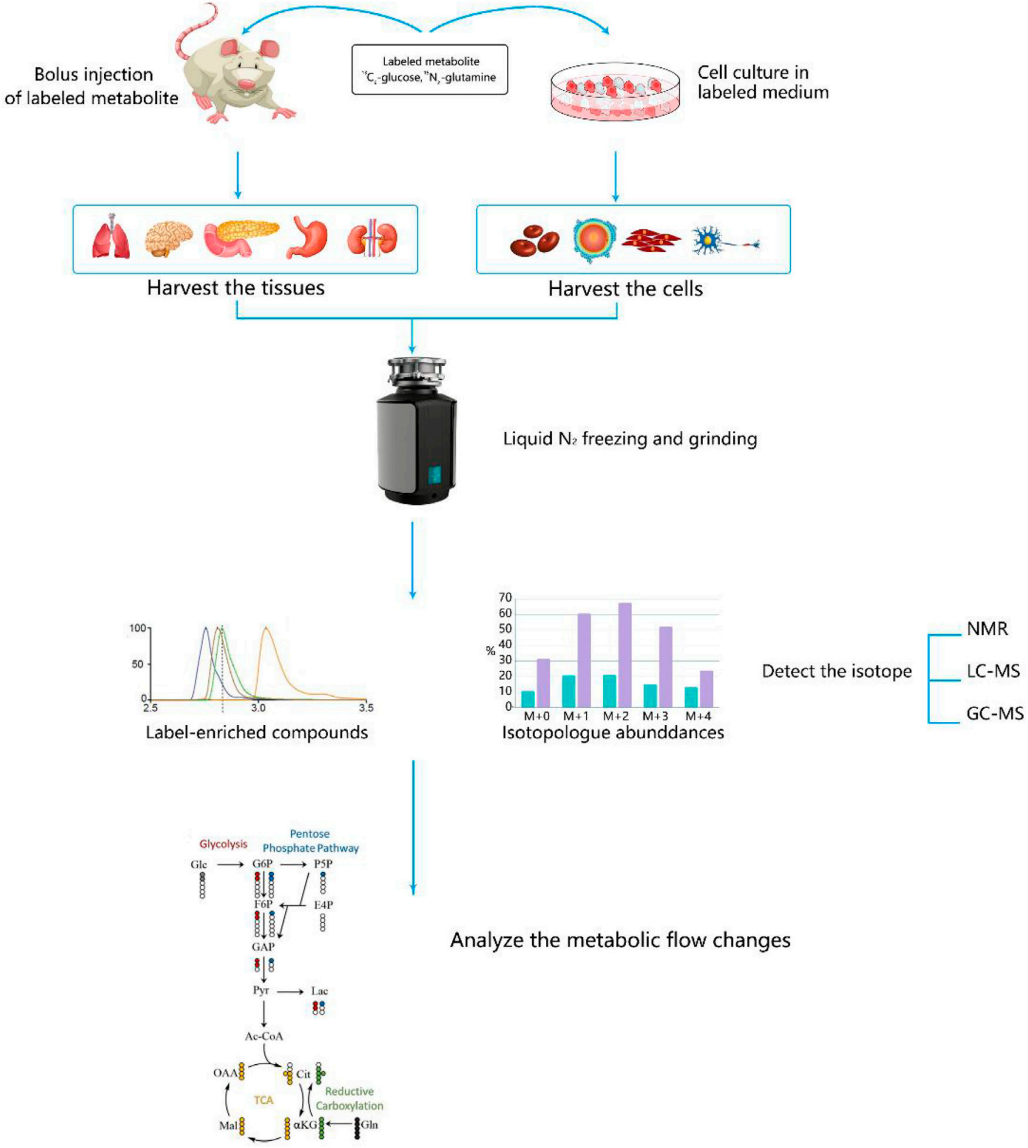
Steps for studying cancer metabolism using stable isotope-resolved metabolomics (SIRM). Stable isotope tracers, such as uniformly ^13^C-labeled glucose (^13^C_6_-Glc) or uniformly ^13^C,^15^N-labeled glutamine (^13^C_5_,^15^N_2_-Gln), are administered via addition to the culture medium for cells or via intravenous injection into whole organisms. Metabolites are then extracted, labeled by tracers, and subjected to various NMR and MS analyses to probe metabolic activity.

Quantitative analysis of metabolism has improved our understanding of metabolic features including metabolite concentrations, fluxes, and free energies. With the development of nuclear magnetic resonance, mass spectrometry, and other technologies, the application of stable isotope tracer technology to the field of metabolomics has become an important aspect of biological research (Bruntz et al., 2017). Stable isotope-resolved metabolomics (SIRM) is a method that extrapolates metabolic pathways and fluxes via the analysis of changes in stable isotope tracer precursor substances to substances. SIRM works primarily by injecting isotopically-enriched precursors, such as [^13^C6]-Glc, into biological systems and detecting subsequent metabolic transformations. Incorporating stable isotopes, such as 2H, 13C, or 15N, into biological precursors has long been used to trace their metabolism in living systems. . Biological samples can be studied using NMR, mass spectrometry, and other detection platforms (Fan et al., 2012) (Figure 3).

Glucose plays a vital role in important glucose catabolic pathways, such as the glycolysis and TCA cycles; thus, ^13^C-labeled glucose is commonly used as a tracer. Metabolic differences derived from SIRM have shown that energy and anabolism are increased in cultured lung cancer cells and NSCLC compared with those in the normal lung (Lane et al., 2011). Moreover, a study using SIRM found that the proliferation of cancer cells can be reduced by inhibiting the glycolysis pathway in cancer cells (Gu et al., 2016). Researchers used ^13^C-labeled glucose to track the glycolysis pathway in lung cancer patients and animal models (Hu et al., 2019) and revealed that lactic acid contributes more to the Krebs cycle than does glucose. This result suggests that lactic acid is the end metabolic waste of the Warburg effect and provides new opportunities for the diagnosis and treatment of cancer (Hui et al., 2017). Proliferating cells shunt glucose into pathways other than the glycolysis and Krebs cycle pathways, and SIRM has confirmed that an increase of glucose flux into these pathways occurs. For example, enhanced non-oxidative and oxidative pentose phosphate pathway activity has been reported in pancreatic and renal cancers, respectively, using a (^13^C2-1, 2)-glucose tracer (Boros et al., 2005; Yang et al., 2013). In addition, many clinically successful drugs and promising candidates for drug targeting tumor therapy may benefit from SIRM analyses to gain insights into their molecular mechanisms. For example, SIRM and microarray experiments have demonstrated that selenium agents perturb Krebs cycle activity and attenuate lipid biosynthesis in lung cancer cells, and these alterations are related to the activation of the AMP-activated protein kinase pathway. Anti-cancer target discovery is one of the most promising translational applications for SIRM, and inhibitors of several targets have been developed and are showing promise in preclinical models (Svensson et al., 2016).

In recent years, Dynamic nuclear polarization enhanced magnetic resonance imaging based on isotope tracer have become dependable imaging tools for the diagnosis and treatment assessment in cancer. Based on preclinical studies that have demonstrated the use of hyperpolarized (1–13C] -pyruvate imaging tools for prostate cancer, Researchers have investigated the *in vivo* pharmacokinetics and pharmacodynamics of hyperpolarized (1–13 C)-pyruvate in order to apply as a tool for imaging liver cancer (Salamanca-Cardona and Keshari, 2015). Applications of probes other than pyruvate are still in the early stages, but molecular imaging of real-time metabolic events could be a valuable tool to elucidate hitherto undiscovered metabolic fluxes that play a role in cancer development and treatment (Perkons et al., 2021).

By gaining insights into metabolic dysfunction due to cancer development or drug interventions, Metabolic flux analysis and fluxomics can be integrated with genomic and proteomic information to achieve systems biochemical insights in both model systems and individual human patients.

## Summary and Future Directions

Metabolomics has been used across many aspects of cancer research, which include cancer pathophysiology, biomarker discovery, and therapeutic response. Metabolic reprogramming is a hallmark of malignancy, and changes in metabolic profiles strongly influence cancer development, progression, and response to treatment. Metabolomics techniques can be used to monitor the dynamics of tumor metabolism and response to treatment over the course of the disease. Moreover, another area of increasing importance is the identification of biomarkers for personalized treatment strategies. At the same time, metabolomic analysis may also yield more accurate and useful clinical information about the metabolic needs of the tumor, as well as the identification of new pharmacodynamic biomarkers and the monitoring of drug resistance of the tumor. On this basis, the applications of stable isotope tracer technology to the field of metabolomics have become an important part of biological research to track the activity of metabolites in the body and trace deep metabolic pathways.

However, there are still several limitations in the study of metabolic reprogramming in tumors using metabolomics. Methodologically, absolute homogeneity across different batches of biological samples cannot be achieved, and the techniques and methods used should be optimized to establish a set of highly sensitive routine procedures that apply to metabolites of varying polarity across different samples. Secondly, most existing studies focus on the metabolomics of biological fluids or extracellular metabolites; however, it is also of importance to study the metabolic characteristics of intracellular metabolites and cancer cells. Thirdly, the mechanisms underlying metabolic changes need to be clarified. This can be achieved by integrating transcriptomic and/or proteomic analyses to identify genes or proteins that cause or are associated with metabolomic changes, which may be potential targets for tumor therapy. Analyzing metabolic changes in tumor cells in response to drugs and revealing the metabolic mechanism underlying tumor drug resistance may provide opportunities to overcome tumor chemotherapy resistance or reverse tumor sensitivity via metabolic regulation. Metabolomics provides a novel approach to study the metabolic reprogramming of tumors and will continue to be widely used in the diagnosis and treatment of different tumors in the future.
